# The essential role of complement in antibody‐mediated resistance to *Salmonella*

**DOI:** 10.1111/imm.13000

**Published:** 2018-10-10

**Authors:** Omar Rossi, Chris Coward, Yun Shan Goh, Jill W. C. Claassens, Calman A. MacLennan, Sjef J. Verbeek, Pietro Mastroeni

**Affiliations:** ^1^ Department of Veterinary Medicine University of Cambridge Cambridge UK; ^2^ Singapore Immunology Network Agency for Science, Tecnology and Research Singapore Singapore; ^3^ Department of Human Genetics Leiden University Medical Center Leiden The Netherlands; ^4^ Nuffield Department of Medicine Jenner Institute University of Oxford Oxford UK; ^5^ Present address: GSK Vaccines Institute for Global Health Siena Italy; ^6^ Present address: Summit Therapeutics Abingdon UK; ^7^ Present address: Department of Biomedical Engineering Toin University of Yokohama Yokohama Japan

**Keywords:** C3, complement, Fc*γ*R, *in vivo*, infection, *Salmonella*

## Abstract

Vaccines can serve as essential tools to prevent bacterial diseases *via* the induction of long‐lasting IgG responses. The efficacy of such vaccines depends on the effector mechanisms triggered by IgG. The complement system and Fc‐gamma receptors (Fc*γ*Rs) can potentially play a crucial role in IgG‐mediated immunity against bacterial diseases. However, their relative importance *in vivo* is unclear, and has been the object of controversy and debate. In this brief study, we have used gene‐targeted mice lacking either Fc*γ* RI, II, II and IV or the C3 complement component as well as a novel mouse strain lacking both C3 and Fc*γ*Rs to conclusively show the essential role of complement in antibody‐mediated host resistance to *Salmonella enterica* systemic infection. By comparing the effect of IgG2a antibodies against *Salmonella* O‐antigen in gene‐targeted mice, we demonstrate that the complement system is essential for the IgG‐mediated reduction of bacterial numbers in the tissues.

## Introduction

Bacterial diseases cause approximately six million deaths per year, and vaccines are key tools to prevent infections.^1^ IgG are generated upon vaccination following class switching and maturation of the humoral response, and represent the main type of antibodies found in blood and extracellular fluids. IgG bind to bacteria and can either enhance uptake by phagocytic immune cells via Fc‐gamma receptors (Fc*γ*Rs), or can trigger the activation of the complement system resulting in: (i) the lysis of the bacterium; or (ii) the deposition of complement fragments on its surface followed by recognition mediated by complement receptors on the phagocyte surface,^2^ resulting also in uptake of the bacterium.

Defining the mechanisms by which antibodies protect against infection is central to the design of new vaccine formulations, new adjuvants and delivery systems, and to understanding of the impact of widespread immune‐suppressive conditions [e.g. malaria, human immunodeficiency virus (HIV)] that can alter the function of immune cells, and affect the antibody and complement system.^3^

The relative importance of complement and Fc*γ*Rs in antibody‐mediated host resistance to many bacterial infections including *Salmonella* diseases has been controversial and difficult to determine with discrepancies between different species and experimental systems.^4^, ^5^, ^6^, ^7^, ^8^, ^9^ This has made it difficult to understand the *in vivo* mechanisms that underpin antibody‐mediated antimicrobial functions and, moreover, has not allowed the identification of reliable and representative protocols in animal experimental systems that could be used to assist human vaccinology.

In mice, antibody‐dependent resistance has until now been inferred not to involve complement. In fact, protection against oral infection in immunized animals, which is IgG dependent,^10^ does not require the C3 component of complement, but is dependent on the function of Fc*γ*RI, II and III.^4^ Moreover, uptake and killing of IgG‐opsonized *Salmonella* by murine macrophages and human phagocytic cell lines can be mediated by Fc*γ*Rs in the absence of complement.^5^, ^6^, ^7^ However, poor complement activity has been inferred to be the cause of the poor antibody‐dependent bactericidal activity of mouse serum against non‐typhoidal strains of *Salmonella*.^8^ Contrary to what is seen in the mouse and in human cell lines, complement appears to play an essential role in antibody‐mediated uptake and killing of *Salmonella* by human primary blood phagocytes and in the activation of their respiratory burst.^9^

In the present study, we have used an intraperitoneal challenge model in a novel mouse strain lacking simultaneously all Fc*γ*Rs and the C3 component of the complement system to dissect *in vivo* the impact of Fc*γ*R and of complement in the control of a bacterial infection. Here we show unequivocally that C3 plays an essential role in IgG2a‐mediated host resistance to *Salmonella in vivo*.

## Materials and methods

#### Mice

Three sets of gene‐targeted animals (referred to as Fc*γ*R KO, Fc*γ*R/C3 KO, and C3 KO in the text) and wild‐type (WT) control animals, all in C57/BL6 background, have been used in this study.

C57BL/6 Fc*γ*RI^–/–^ Fc*γ*RII^–/–^ Fc*γ*RIII^–/–^ Fc*γ*RIV^–/–^ mice (Fc*γ*R KO mice) lack simultaneously Fc*γ*RI, Fc*γ*RII, Fc*γ*RIII and Fc*γ*RIV, and were generated in the transgenic mouse facility of the Leiden University Medical Center.^11^ C57BL/6 C3^–/–^ gene‐targeted mice (C3 KO mice) were kindly provided by Dr Mike Carroll (CBR Institute for Biomedical Research, Harvard Medical School, Boston, USA); they lack the C3 component of complement,^12^ thus are unable to activate the classical and alternative complement pathways. C57/BL6 Fc*γ*RI^–/–^ Fc*γ*RII^–/–^ Fc*γ*RIII^–/–^ Fc*γ*RIV^–/–^ C3^–/–^ (Fc*γ*R/C3 KO mice) lack simultaneously Fc*γ*RI, Fc*γ*RII, Fc*γ*RIII, Fc*γ*RIV and C3, and were obtained by crossing the Fc*γ*R KO animals with the C3 KO mice. All groups were matched for strain, age and sex. Gene‐targeted mice were bred at the laboratory animal facility (PDC) of the Leiden University Medical Center, the Netherlands, and shipped to the animal facility of the University of Cambridge 2 weeks before the start of the experimental procedures. WT C57/BL6 mice were purchased from Envigo UK. The health status of the animals was monitored over time. Animals tested negative for all agents listed in the FELASA (Federation of European Laboratory Animal Science Associations) guidelines for SPF mouse colonies.^13^

#### Bacterial infections and passive transfer of IgG

Anti‐O4 IgG2a monoclonal antibodies^14^ and IgG2a isotype controls (Southern Biotech) were diluted in sterile phosphate‐buffered saline (PBS; Sigma Aldrich, St Louis, Missouri, United States) at a final concentration of 0·5 mg/ml; 0·1 mg of antibodies was passively transferred to the mice by intravenous (i.v.) injection into a lateral tail vein 12 hr before infection.

*Salmonella enterica* serovar Typhimurium (*S*. Typhimurium) SL1344,^15^ a virulent strain with an i.v. LD_50_ < 20 Colony Forming Units (CFU) for C57BL/6 mice, was used for infections. Bacteria were grown statically in Luria–Bertani (LB) broth at 37° for 16 hr before being diluted in sterile PBS. *S*. Typhimurium (~1 × 10^5 ^CFU) was injected intraperitoneally (i.p.) into each animal. Twenty‐four hours after infection mice were killed by cervical dislocation. Spleens, livers and mesenteric lymph nodes (MLNs) were removed and individually homogenized in sterile water (5 ml for livers and spleens, 2 ml for MLNs) using a Stomacher 80 (Seward) homogenizer. Homogenates were plated onto LB agar.

#### Evaluation of early events in the peritoneal cavity

Bacteria were grown statically for 16 hr at 37° in LB broth. Opsonization was performed by incubating bacteria at ~10^8 ^CFU/ml with 6·75 μg/ml of anti‐O4 monoclonal IgG2a, or with isotype control IgG2a, at 37° with shaking for 30 min; non‐bound antibodies were removed by washing with PBS. The optimal concentration of anti‐O4 IgG2a for opsonizing bacteria was determined as the lowest dilution that would not cause bacterial agglutination.^7^ Effective opsonization was visualized with fluorescence microscopy using Alexa Fluor 568‐conjugated goat anti‐mouse IgG (Invitrogen, Paisley, UK).

Mice were injected i.p. with ~5 × 10^6^ CFU of opsonized bacteria. Thirty minutes after infection, peritoneal washes were collected *via* lavage using 5 ml ice‐cold PBS supplemented with 2% heat‐inactivated foetal bovine serum (Life Technologies, Carlsbad, California, United States) and 4 mm EDTA (Sigma Aldrich). Bacteria were enumerated by counting appropriate dilutions in LB agar after treatment of the lavage fluid with 0·1% Triton X (Sigma Aldrich) for 15 min to release intracellular bacteria.

#### Ethics

All animal experiments were performed at the University of Cambridge in accordance with good animal practice as defined by the relevant international (Directive of the European Parliament and of the Council on the Protection of Animals Used for Scientific Purposes, Brussels 543/5) and local (University of Cambridge) animal welfare guidelines. This research has been regulated under the Animals (Scientific Procedures) Act 1986 Amendment Regulations 2012 following ethical review by the University of Cambridge Animal Welfare and Ethical Review Body (AWERB).

#### Statistical analysis

To compare the effect of passive transfer of anti‐O4 IgG2a on log_10_(CFU) recovered from the different groups of mice, the Conover *post hoc* method for multiple comparisons was applied to the data using the Holm–Bonferroni technique to adjust the *P*‐values. Comparisons between all the experimental groups were also performed by the Mann–Whitney test (two‐tailed) to the 28 possible pairings of the eight groups.

To compare the relative differences in terms of log_10_(CFU) values observed between anti‐O4 IgG‐treated and isotype control‐treated animals of one mouse strain (*X*_Δ_) with those observed between anti‐O4 IgG‐treated and isotype control‐treated animals of another mouse strain (*Y*_Δ_), all the possible comparisons of empirical distributions *X*_Δ_ and *Y*_Δ_ have been tested using the two‐sample Kolmogorov–Smirnov test with respect to the null hypothesis that *X*_Δ_ and *Y*_Δ_ are randomly drawn from the same probability distribution.

All statistical tests have been performed at a 5% significance level (*P *< 0·05) using R version 3.3.

## Results

### The relative importance of C3 and Fc*γ*Rs in antibody‐mediated resistance to a systemic *Salmonella* infection

Groups of six WT mice, six mice lacking either Fc*γ*RI, II, III or IV (Fc*γ*R KO), six mice lacking the complement C3 component (C3 KO), and six mice lacking both Fc*γ*R and C3 (Fc*γ*R/C3 KO) were passively immunized with anti‐O4 IgG2a monoclonal antibodies.^14^ Control animals from each strain received IgG2a isotype control antibodies. All mice were infected i.p. with ~1 × 10^5 ^CFU of *S*. Typhimurium SL1344.

**Figure 1:**
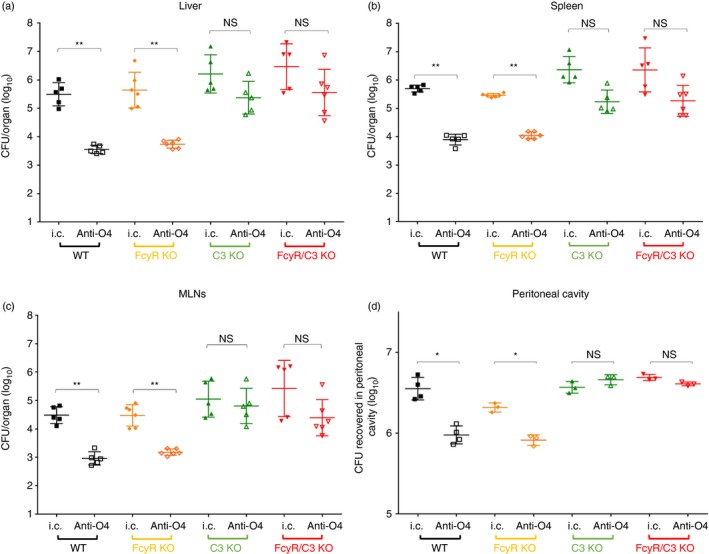
Bacterial loads recovered from different organs. (a–c) Different groups of animals received IgG2a (anti‐O4, or isotype control – i.c.) 12 hr before being infected intraperitoneally (i.p.) with 10^5^ Colony Forming Units (CFU) of *Salmonella enterica* serovar Typhimurium (*S.* Typhimurium). (a) Bacterial loads in liver 24 hr after infection. (b) Bacterial loads in spleen 24 hr after infection. (c) Bacterial loads in mesenteric lymph nodes (MLNs) 24 hr after infection. (d) Bacteria were opsonized *in vitro* with anti‐O4 IgG or treated with the same concentration of isotype control antibodies prior to being injected i.p.; 30 min later bacterial loads were determined in peritoneal washes. Each symbol represents total CFU count from an organ of a single mouse; horizontal line shows the mean ± standard deviation on each experimental group. *P < 0·05, **P < 0·01, NS, not significant. Results shown are representative of two experiments.

Viable bacteria were enumerated in homogenates of the liver (Fig. 1a), the spleen (Fig. 1b) and the MLNs (Fig. 1c) 24 hr after infection. Passive immunization with anti‐O4 IgG2a reduced bacterial numbers in the tissues of WT mice and Fc*γ*R KO mice by ~2‐log_10_ CFU compared with animals treated with control IgG2a. In contrast, both in C3 KO and in Fc*γ*R/C3 KO animals, although the variance in recovered CFU from organs in these groups is higher compared with mice lacking only Fc*γ*R or WT animals, passive immunization did not result in a significant reduction in the bacterial loads in the liver, spleen and MLNs.

This indicates that C3, but not Fc*γ*R, is necessary for IgG2a‐mediated antibacterial function of the mouse immune system following i.p. challenge.

### Bacterial killing in the peritoneal cavity

The inactivation of *S*. Typhimurium within the peritoneal cavity was explored by measuring the amount of viable bacteria recovered from peritoneal washes 30 min after i.p. injection of bacteria pre‐opsonized *in vitro* with anti‐O4 monoclonal IgG2a or with control IgG2a.

Administration of *S*. Typhimurium pre‐opsonized with anti‐O4 IgG2a resulted in a reduction in the viable bacterial numbers harvested from the peritoneal cavity of WT mice and Fc*γ*R KO mice. Conversely, pre‐opsonization had no significant effect on the amount of bacteria recovered from the peritoneum of C3 KO and Fc*γ*R/C3 KO animals (Fig. 1d).

The reduction in the recovery of anti‐O4 IgG2a‐opsonized viable bacteria in the peritoneal cavity of Fc*γ*R KO mice was significantly less than what observed in WT mice (*P *< 0·01), showing that when mice are injected i.p., Fc*γ*Rs play a detectable, albeit minor, role in host resistance. No significant difference in the IgG2a‐mediated inactivation of bacteria in the peritoneal cavity was seen between Fc*γ*R/C3 and C3 KO animals (*P *= 0·99), showing no additive effect on the reduction of bacterial numbers in the peritoneum due to the simultaneous lack of C3 and Fc*γ*R.

## Discussion

The availability of mice lacking all four Fc*γ*R plus the C3 component of complement has enabled us to directly compare the relative importance of Fc*γ*R and the complement system in IgG‐mediated host defence against *Salmonella* infections. We used IgG2a in this study as this subclass has strong binding affinity for all Fc*γ*R, including as a monomeric IgG and it’s a powerful complement activator.^16^

We showed that both in C3‐deficient and in Fc*γ*R/C3‐deficient animals, anti‐*Salmonella* IgG2a was unable to induce a reduction in the number of viable bacteria in the spleen, liver, MLNs and peritoneal cavity. In contrast, the absence of Fc*γ*R had little effect on the antimicrobial function of IgG.

The results shown in this brief report conclusively establish the so‐far elusive and controversial role of complement in IgG‐mediated antimicrobial resistance *in vivo* in the mouse. Complement is required for antibody‐dependent killing of *Salmonella* by human blood phagocytes^9^ and in the absence of cells (antibody‐dependent complement‐mediated killing).^17^ Therefore, the demonstration that complement is also required for antibody‐mediated killing of *Salmonella* in mice lends support to the study of infection in mice as a model of invasive *Salmonella* disease in man, and possibly of other bacterial infections.

The mechanisms by which complement mediates IgG‐induced host resistance to i.p. challenge remain to be elucidated. It is likely that cell‐mediated effector mechanisms are involved, given the reported inability of mouse complement to mediate IgG‐induced serum bactericidal activity against wild‐type *Salmonella* strains.^8^

Our previous work has shown that complement does not play an essential role in the resistance against oral challenge with virulent *Salmonella* in mice immunized with live attenuated vaccines. Despite the known^10^ requirement for antibodies in protection in this model, the lack of Fc*γ*RI, II and III, but not C3, resulted in impaired resistance in the vaccinated animals. These results illustrate that the contribution of the two downstream components of antibody effector mechanisms, Fc*γ*R and complement, varies between models. Although the i.p. challenge model is not the natural route of infection, it may be more representative than the oral route when modelling invasive *Salmonella* disease in man. We focus our attention only on IgG2a in this study because this subclass has strong binding to all Fc*γ*Rs, including as a monomeric IgG, and it is also a powerful complement activator.^16^

The differences in the bacterial numbers seen in the spleen, liver and MLNs are likely to be a consequence to the early events in the peritoneal cavity before the bacteria colonize the systemic compartment. The requirement for complement in the IgG‐mediated reduction in bacterial numbers can be observed in the peritoneal washes 30 min after inoculation. Interestingly and differently from what is seen in the systemic compartment, the effect of IgG‐mediated bacterial killing in the peritoneal cavity of animals lacking Fc*γ*R is lower than that observed in WT animals, indicating a small but significant contribution of Fc*γ*R.

In conclusion, the elusive *in vivo* role of complement in the antimicrobial function of IgG against virulent *Salmonella* can be demonstrated using an i.p. challenge model. This mirrors the dependence on complement of killing *Salmonella* in human blood.^9^, ^17^

## Author contributions

ORo, SJV, PM conceived and designed the experiments; ORo, CC, YSG, JWCC, PM performed the experiments; ORo, PM analysed the data; YSG, SJV, CAM provided reagents and tools; ORo, Cc, YSG, JWCC, CAM, JSV, PM contributed to the writing of the manuscript.

## Disclosures

The authors have the following conflict of interest to declare: ORo is currently an employee of the GSK Vaccines Institute for Global Health, part of the GSK group of companies. CC is currently an employee of Summit Therapeutics plc. YSG and CAM for part of the time in which the study was conducted were employees of Novartis Vaccines Institutes of Global Health. This does not alter the authors’ adherence to all Journal policies on data and material sharing.
